# Meeting report: microbes as safeguards of the environment

**DOI:** 10.1093/sumbio/qvae013

**Published:** 2024-05-24

**Authors:** Juan L Ramos, Víctor de Lorenzo, Puri López

**Affiliations:** Estación Experimental del Zaidín, CSIC, Profesor Albareda 1, 18008 Granada, Spain; Systems Biology Department, Centro Nacional de Biotecnología, CSIC C/ Darwin, 3 Madrid-Cantoblanco 28049, Spain; Ecologie Systématique Evolution, CNRS, Université Paris-Saclay, AgroParisTech, 91190 Gif-sur-Yvette, France

**Keywords:** climate change, plant growth promoting rhizoorganisms, microbes, biogeochemical cycles, end hunger, eutrophication, bioenergy, xeobiotic compounds, zoonosis, synthetic biology

## Abstract

From 12 to 14 March, a gathering of microbiologists and biotechnologists convened at the International University of Andalucía (UNIA) in Baeza (Jaén, Spain) under the auspices of UNIA and the Applied Microbiology International (AMI) Society. The primary objective of this meeting was to analyse the pivotal role microbes play in sustaining our planet in a broader context—both from the descriptive (what is the state of affairs) and the prescriptive perspective (what to do and look for regarding activities of interest). The workshop focused on exploring the ecological aspects of microbes in soil and water, including some extreme environments, the potential of microorganisms as promoters of plant growth and biocontrol agents as well as active large-scale catalysts for environmental health. Various topics were examined in this context, encompassing the application of microbes as platforms for the biosynthesis of value-added chemicals, bioremediation technologies, the concept of the circular economy, the emergence of zoonotic concerns within a dynamically changing global environment, and the role of microbes in precision nutrition and precision medicine. In alignment with the strategic plan of AMI, the discussion was focused on the background of the United Nations Sustainability Development Goals (UN SDGs). These targets were formulated at the beginning of the past decade to guide humanity towards a sustainable future. The UN SDGs aim to prevent the overstepping of planetary boundaries, which are endangering Earth’s biodiversity and even human survival.

Sustainability Statement Microbes are the most abundant and diverse life forms on Earth. They play a crucial role in biogeochemical cycling and have potential to mitigate climate change. However, they are often overlooked in models and discussions on environmental challenges. There is a growing advocacy for urgent actions to integrate microbiology into global initiatives, such as the UN Sustainable Development Goals (SDGs), to fully harness their potential. Understanding microbial networks is essential for ecosystem functionality and planetary sustainability. Microbes contribute to most of the SDG in various ways: nurturing health and addressing diseases (SDG 3), food production and nutrition (SDG 2), production of clean energy (SDG 7), synthesizing and recycling chemicals, removal of pollutants, clean water (SDG 6), and global biochemical cycles. Microbes have immense potential as allies in addressing contemporary threats to our planet.

## Introduction: the Earth is a microbial planet

Viewed from a geological and biological standpoint, our planet is a living entity. The accretion of the Earth is estimated to have occurred ∼4.6 billion years ago, evolving with a hot nucleus, mantle, and crust—the latter hosting all forms of life. The Earth’s crust spans diverse environments, from the depths of oceans to mountains, rivers, freshwater, highly saline, low- or high-pH lakes, and estuaries, along with prairies, tropical forests, and extremely cold poles, collectively shaping the Earth’s landscape. This geographic diversity results in a wide range of temperatures, humidity levels, and radiation gradients, which define as many biotopes.

Life in its simplest forms emerged around 3.5–3.8 billion years ago, probably started by some sort of protocells (Sanchez-Baracaldo et al. 2022) that evolved into prokaryotic-like ancestors. Over time, their derivatives, archaea, and bacteria have successfully colonized every possible niche on the planet, adapting to widely different physicochemical conditions and evolving incredibly diverse metabolic capabilities. Cyanobacteria, able to derive electrons from water photolysis and release molecular oxygen as a waste product, Rassmusen et al. (2009) played a pivotal role in evolving the efficient oxygenic photosynthesis that accounts for most current primary production and shaping the current oxic atmosphere. Furthermore, cyanobacteria and other photosynthetic bacteria have contributed to massive carbonate deposition over geological history (Sheehan et al. 2004, Falkowski et al. 2008, Dupraz et al. 2009, Benzerara et al. 2022). The oxygenation of the atmosphere around 2.4 Ga ago probably set the stage for the evolution of the eukaryotic cell and, subsequently, that of complex multi-cellular organisms, such as animals, plants, fungi, and kelp (López-García and Moreira. 2023). For most of the Earth’s history, though, life was microbial (Bertrand et al. 2015). Microbes represent the widest biodiversity in our planet, with archaea, bacteria, and microbial eukaryotes harbouring the widest genetic and metabolic diversity. *Homo sapiens* appeared only around 300 000 years ago. Whilst humans coexisted in equilibrium with their environment for millennia, their adaptive cognitive capacities, with the development of language and communication, the use of fire and progressively engineered tools, led to their growing impact on ecosystems and the natural course of the Earth’s history. The late XVIII century marked the onset of the industrial era, which had more far-reaching consequences on our species impact on our planet than the previous thousands of years of human evolution, triggering climate change and biodiversity loss at unprecedented rates (Merz et al. 2023).

Life on our planet is dynamic and takes place within a semi-closed system, with the Earth receiving sunlight and some extra-terrestrial matter, essentially in the form of meteorites, from the Solar System. It is believed that most water on Earth was brought by meteorites during the early Earth. Water is essential for life. The foundation of life is based on the continuous cycling of biogenic elements—such as carbon, nitrogen, sulphur, and phosphorus as the main nutrients and constituents of all living organisms—alongside magnesium, calcium, iron, and various other metals acting as catalysts in numerous reactions. Unfortunately, the overexploitation of non-renewable resources, particularly fossil fuels, has triggered a rapid release of gases with a greenhouse effect, as well as many pollutants. These emissions contribute to pressing global challenges, including planet-wide increases in temperatures, severe droughts, widespread wildfires, and extensive flooding (IPCC 2014, 2019, Betts et al. 2023, Merz et al. 2023, Ripple et al. 2023). These issues are behind the current loss of biodiversity, ocean acidification, zoonotic diseases, and other adverse effects, all ultimately connected to human behaviour, in particular industrial, agricultural, and urban activities (Ripple et al. 2022).

## Microorganisms are key players for dealing with SDGs

Given that microbes are the most diverse and abundant forms of life on this planet and that they are ultimately responsible for biogeochemical cycling, they also possess the potential to become our best allies in mitigating, and even solving, some of the serious contemporary threats to life on Earth (Anand et al. 2023). Yet, whilst microorganisms are undoubtedly pivotal players for achieving the United Nations Sustainability Development Goals (UN SDGs), they seem to be most often ignored in virtually all models and equations dealing with climate change (Bourzac 2023, Gewin 2023, Singh et al. 2023a, 2023c). This must be changed urgently, as the environmental microbiome, because of its extension and activity, might be the only realistic asset that we have for bringing about the global transformations that are so badly needed.

To engage microbes as guardians of the planet, a profound understanding of their properties is needed, and education emerges as the critical factor. UN SDG4 endeavours to ensure universal access to primary and secondary education, a goal still far from realization. The United Nations emphasizes the importance of enhancing literacy, numeracy, and basic mathematical skills worldwide. A key challenge in education lies in ensuring an adequate number of qualified teachers and reaching individuals in remote locations. Addressing these challenges necessitates exploitation of the Internet to disseminate knowledge globally. Microbiology training can play a crucial role in secondary and high school education, as showcased by the International Microbial Literacy Initiative (iMiLi), led by Ken Timmis and involving nearly 1000 scientists worldwide (https://www.imili-eah.com/, Timmis 2023b). At the Baeza workshop, Ken Timmis emphasized the significance of incorporating microbiology into secondary and high school curricula if we want young people and the leaders of future generations to leverage the potential of microbes for a better tomorrow. The iMiLi initiative seeks to raise awareness about the vital roles microbes play in sustaining healthy soil, cleaning polluted waters, and influencing the human microbiome, which impacts health and nutrient assimilation (Timmis 2023a, 2023b). Microbial networks form the foundation of the many interactions that ultimately dictate ecosystem functionality and thus planetary sustainability. Knowing the behaviour and capacities of the global microbial realm is as important as understanding global economics or geopolitical frames.

## Microbes for combatting hunger and eradicating poverty

The UN SDG 2 aims at eradicating hunger, i.e. ‘*End Hunger’*, a goal of paramount importance as our global population is anticipated to reach 9 billion individuals by 2050. The 2023 United Nations Report on SDG Advances revealed that 735 million people are suffering chronic malnutrition along with frequent episodes of famine (UN report 2023). To counteract this, we need not only more and better food, but also new ways of production and access. COP28 has highlighted significant planetary changes originating from fossil fuel use with high levels of pollution and heightened levels of CO_2_ and methane as greenhouse gases that are contributing to the Earth’s warming. A number of experimental studies have indicated that elevated CO_2_ levels (e.g. >450 ppm) and increased temperatures (2 degrees above the pre-industrial era) will not only diminish plant productivity but also compromise food quality, particularly in terms of protein content rich in essential amino acids (Moore et al. 2021). In this evolving global landscape with an increasing population and a higher demand for high-quality food, sustainable production practices become imperative (Bernal 2024). Whilst the Green Revolution of the past century boosted crop yields through intensive fertilizer and pesticide use, it has proven detrimental to the environment, causing soil pollution, water eutrophication, the impoverishment of soil microbiomes, and a dramatic decline of pollinator populations. Simultaneously, large-scale deforestation in regions like the Amazon and South Asia not only diminishes biodiversity but also triggers soil erosion and thus less productivity. One key approach for addressing the need for environmentally friendly primary production involves leveraging microbes that interact with plants, promoting nutrient mobilization, and stimulating plant growth through the production of hormones or their precursors as discussed by Patricia Bernal (2024). Brajesh Singh, Miguel Matilla, and María-José Pozo described how many of the plant growth-promoting microbes safeguard plants against pathogens by producing natural antifungal and antibacterial compounds and sequestering iron, thus limiting pathogen growth (Pozo de la Hoz et al. 2021, Matilla et al. 2022, Roca and Matilla 2023, Singh et al. 2023a, 2023b, 2023c). Another crucial aspect of ending hunger is minimizing food losses, both in the field during cultivation and post-harvest (Sun et al. 2022, Bernal 2024).

Crop yields are intricately linked to climate conditions and water availability. Therefore, concerted efforts in establishing infrastructures that ensure water for crops, without disrupting or minimally affecting the water flow along rivers and estuaries, are essential for sustainability. Brajesh Singh featured discussions on engineering microbial communities, focusing on simulating the plant microbiome and using symbiotic consortia (notably bacteria and mycorrhiza) to foster robust plant growth (Singh et al. 2023a, 2023b, 2023c).

The pursuit to end hunger aligns directly with UN SDG 1, which aims to eradicate poverty, and UN SDG 3, promoting well-being. Achieving this goal demands an increase in the microbiological literacy of farmers in both developed and developing nations. Such training should not only emphasize the use of soil as a support for plant growth, but also encourage the exploitation of all kinds of soil through judicious land management to optimize nutrient utilization and promote natural biogeochemical cycling (Timmis and Ramos 2021). It is time to unite plants and microbes to enhance productivity and eliminate hunger—rather than exclusively depending on chemical fertilizers and genetically modified crops.

## Nurturing catalysts for a cleaner environment

As mentioned above, COP28 has unequivocally recognized the pollution challenges derived from the widespread use of fossil fuels and, for the first time, has articulated mandates to transition to more sustainable forms of energy (note that United Nations recommendations are not compulsory for its member states or the signatories of Treaties). Regarding energy, COP28 promotes decarbonization through use of alternative energies such as wind, thermosolar, photovoltaics, and marine wave energy. Microbes emerge as valuable partners in the journey towards zero emissions from energy consumption. Bioenergy initiatives, initially led in the USA and Brazil to diminish reliance on gasoline, have seen blends with ethanol of up to 10% in use (Somerville et al. 2010, Valdivia et al. 2016). The European Union has also embraced these initiatives more recently. The use of first-generation biofuels, derived from grains, opened up a debate over the trade-off between food and fuel, prompting the development of second-generation biofuels that utilize non-edible agricultural residues and organic waste. Organic waste is also a source of biogas (mainly methane) produced in bioreactors by methanogenic archaea in complex communities. A diversified energy mix is the solution to phase out fossil fuels and bioenergy stands poised to curtail emissions linked to transportation and chemical factories. One of the by-products of second-generation biofuels derived from agricultural waste is a lignin-rich cake, traditionally burned for energy, which represents not only a source of CO_2_ emissions but also the loss of a valuable reservoir for synthesizing new (bio)chemicals (Salvachúa et al. 2020, del Cerro et al. 2021, Bele et al. 2023). A number of reports indicate that lignocellulosic residues could be the source to produce up to 70% of commonly used organic compounds, particularly using microbial cell factories. Despite progress, more efforts are needed for enhancing the efficiency of these bioprocesses to achieve large-scale manufacturing and facilitate downstream processing to deliver the products of interest in a way that is competitive with current methods (Ramos and Duque 2019, Keasling et al. 2021, Li et al. 2023). It should also be noted the waste generated from these bioprocesses can still be used to enhance manure and soil amendments, thus contributing to increased fixed carbon. The workshop clearly presented the potential of various microbial platforms as the chassis for producing goods out of very diverse waste products (Cerrone et al. 2023, Hueso-Gil et al. 2023, Wu and Wang 2024).

Whilst hydrocarbon and plastic pollution remain a pressing concern, the use of xenobiotic chemicals in pesticides, household cleaning products, etc. also poses serious pollution problems (Chiba et al. 2018, Michán et al. 2021, Dodge et al. 2024). Larry Wacket at the meeting discussed how certain compounds are highly toxic, either inherently or because their biotransformation yields products that can be incorporated in central metabolites, disrupting proper functioning. Larry Wacket described defluorinases able to remove fluorine from C–F bonds, but fluorine itself represents a serious stress for microbes (Pardo et al. 2022, Dodge et al. 2024). Managing stress imposed by pollutants or their derivatives is essential to prevent and break the loop of ‘forever chemicals’ (Haas and Nikel 2023, Dodge et al. 2024). We need to harness the wide catabolic potential of microbes to combat pollutants either by themselves or in collaboration with plants and, in some cases, leveraging the gut of soil insects and nematodes.

## Towards a (micro) bio-based industry

DG8 and DG9 aim to promote decent jobs, economic growth, and innovation in industry and infrastructure. In the context of these goals, climate change poses a pressing challenge, evident in the global temperature rise of ∼1.5°C compared to the pre-industrial era. The Paris Agreement set a target to limit this increase to below 2°C by 2050, demanding a concerted effort to achieve net-zero emissions. However, achieving this goal remains uncertain, with reports suggesting that it may not be realized before 2070 without substantial and immediate measures (Shears 2019). Whilst societal awareness regarding the risks associated with fossil fuel use is widespread, the chemical industry stands out as the second-largest emitter of greenhouse gases, encompassing petrochemicals and various compounds derived from primary oil-based feedstock. A transformative solution involves the adoption of the concept of bio-factories, capable of producing at least 70% of the world’s chemicals. This includes the production of ammonia from atmospheric nitrogen through the mediation of nitrogen-fixing microbes, hydrogen production in bacterial platforms, and the biological manufacturing of a diverse range of chemicals. The Baeza workshop has underscored the potential of the microbial chassis to generate goods from inorganic compounds and highlighted the transformation of agricultural, industrial, and urban waste into valuable chemicals. Pablo Niket at the meeting discussed the limited range of chemicals found in living organisms compared to the elements on the periodic table. Pablo presented results involving new fluorinase enzymes capable to adding F to C sketetons, which could pave the way for developing new green-chemistry methods (Pardo et al. 2022).

Whilst many questions persist in developing efficient bioreactors and refining product recovery processes, these challenges also present opportunities for creating decent and specialized jobs as well as a very desirable de-centralization of production. Since bio-factories are not geographically limited by the presence of oil or gas reserves, they can be established worldwide (Anand et al. 2023). Along these lines, diverse processes currently under development contemplate the simultaneous generation of multiple chemicals with specific recovery methods for gaseous, liquid, and insoluble products. Also, in this context, the Baeza workshop emphasized the capacity of microorganisms to produce bioplastics and monomers for synthesis of biodegradable polymers with high efficiency, along with a diverse array of chemicals that could replace their industrial counterparts, leading to a remarkable reduction in CO_2_ emissions—up to 90% (Hernández-Arriaga et al. 2022, García-Franco et al. 2024).

Beyond their environmental benefits, adoption of bio-factories (whether *in situ* or *ex situ*) can contribute to addressing the abandonment of rural areas (Anand et al. 2023). Amongst other reasons, they enable the development of new cultivars, utilization of marginal lands, offer incentives for people to settle in more environmentally friendly surroundings, countering migrations, and the trends of moving towards densely populated cities. Advanced bio-factories can also play a role in combating gender inequality, as training becomes foundational for individuals running these innovative businesses. This paradigm creates employment opportunities with gender equality in rural areas, contributing to a reduction in overall societal disparities (Anand et al. 2023).

## Dealing with *bad* microbes and leveraging the *good* ones

According to the annual United Nations report on the progress of the Sustainable Development Goals, the COVID-19 pandemic has prevented advancements towards Goal 3, with one of the most alarming consequences being a decline in childhood vaccinations. The response of governments, international organizations, the scientific community, and pharma companies has played a crucial role in saving lives despite the enduring initial impact. The zoonotic origin of SARS-CoV-19 appears evident, and Harald Brüssow at the meeting in Baeza indicated that a number of analyses suggest that approximately 200 000 viruses may be harboured by mammals, posing a potential risk of interspecies transmission or transfer to avian species, sparking new pandemics (Brüssow 2023). Behind these threats lie the loss of habitats for various wild animals and the repercussions of climate change, including droughts and floods, affecting animal life. In a recent article published in Microbial Biotechnology, Nick Croucher (2024) expressed the need for developing new vaccines against bacteria, not just viruses, and highlighted some challenges in the field. Alongside these developments, there is an ongoing need to persist in vaccinating against diphtheria, tetanus, and whooping cough, as a reported decrease in coverage for the three doses poses a risk to 25 million children worldwide according to United Nations.

Advancements in the knowledge of plants and animal, including human, microbiomes are closely linked to well-being and good health. Beyond the initial exploration of the human gut microbiome and its role in the production of essential amino acids and vitamins, it has become evident that the gut microbiota influences the function of multiple organs such as the brain, heart, and liver—let alone the growing evidence on the gut-brain axis and its role in mental health (Bergstrom et al. 2020). In particular, recognizing that tumours can harbour microbes has led to innovative applications, such as utilizing them as sensors to monitor cancer locations and as potential carriers for drug delivery to combat proliferation or attract immune cells to specifically eliminate given cell types (Nejman et al. 2020). Anticipated massive developments in this field hold promise for important breakthroughs. We anticipate that precision nutrition and microbiome-based medicine will be soon part of standard medical procedures, although they may only be available for a few during their early development (Gilbert et al. 2018, Kazemian et al. 2020, Kalia et al. 2022).

## Towards a fairer interplay with nature

We cannot conclude this report without acknowledging a prevailing sentiment that permeated the Baeza workshop, namely, that we are amidst a pivotal transition in our relationship with the natural world. For millennia, we have regarded nature as something to be subdued, dominated, and exploited solely for our benefit, an attitude that has accelerated since the Industrial Revolution. The repercussions of this longstanding mindset are unmistakably evident today, manifesting in environmental degradation and its consequential impact on food security, mass migrations, and profound inequalities. Whilst many of these pressing issues have roots in socio-political factors, they also possess scientific and evolutionary angles, for which we now possess the necessary tools and fundamental knowledge to address. Particularly compelling is the mounting evidence regarding our intrinsic connection with the broader biological realm. Whilst earlier generations perceived life forms (other than ourselves) as something basically alien to us, we know now that we are essentially linked to them through a shared genetic code and ancestry as well as a continuous, bi-directional microbiome exchange. This should urge us to reassess our interaction with nature not as mere domination, but as a collaborative partnership characterized by dialogue and negotiation with the other biological players of the Biosphere, among which microorganisms stand out as the major actors. In light of this realization, microbes emerge as invaluable allies in preserving the planet as a globally sustainable ecosystem, one that enables the enduring presence of humanity whilst nurturing the well-being and prosperity of its inhabitants.

## Disclaimer

We would like to note that the text above provides insights into the issues analysed during the workshop and is not intended to offer an exhaustive analysis of microbes for the sake of a sustainable world. For further details, the reader is referred to recent publications in the field where more in-depth, specialized information can be obtained (Cavicchioli et al. 2019, Jansson 2023, Anand et al. 2023, Rappuoli et al. 2023).

## Data Availability

All relevant data are contained within this report.

## References

[bib1] Anand S, Hallsworth JE, Timmis J et al. Weaponising microbes for peace. Microb Biotechnol. 2023;16:1091–111. 10.1111/1751-7915.14224 36880421 PMC10221547

[bib2] Bele G, Benali M, Stuart PR. Multicriteria assessment of technology pathways to produce renewable and sustainable biofuels: case study in Eastern Canada. Biofuels Bioprod Bioref. 2023;17:944–60. 10.1002/bbb.2488

[bib3] Benzerara K, Duprat E, Bitard-Feildel T et al. A new gene family diagnostic for intracellular biomineralization of amorphous Ca carbonates by Cyanobacteria. Genome Biol Evolut. 2022;14:evac026. 10.1093/gbe/evac026 PMC8890360 35143662

[bib4] Bergstrom K, Shan X, Casero D et al. Proximal colon-derived O-glycosylated mucus encapsulates and modulates the microbiota. Science. 2020;370:467–72. 10.1126/science.aay7367 33093110 PMC8132455

[bib5] Bernal P. How are microbes helping end hunger?. Microb Biotechnol. 2024;17:e14432. 10.1111/1751-7915.14432 38465536 PMC10926054

[bib6] Bertrand JC, Brochier-Armanet C, Gouy M, Westall F, Bertrand JC (eds) Fundamentals and Applications: Microbial Ecology. Environmental Microbiology. Dordrecht: Springer Science+Business Media, 2015.

[bib7] Betts RA, Belcher SE, Hermanson L et al. Approaching 1.5°C: how will we know we’ve reached this crucial warming mark?. Nature. 2023;624:33–35. 10.1038/d41586-023-03775-z 38036862

[bib8] Bourzac K. Microbiologists at COP28 push for a seat at the climate-policy table. Nature. 2023, 10.1038/d41586-023-03765-1. 38036681

[bib9] Brüssow H. Viral infections at the animal–human interface—learning lessons from the SARS-CoV-2 pandemic. Microb Biotechnol. 2023;16:1397–411. 37338856 10.1111/1751-7915.14269 PMC10281366

[bib10] Cavicchioli R, Ripple WJ, Timmis KN et al. Scientists’ warning to humanity: microorganisms and climate change. Nat Rev Microbiol. 2019;17:569–86. 10.1038/s41579-019-0222-5 31213707 PMC7136171

[bib11] Cerrone F, Zhou B, Mouren A et al. *Pseudomonas umsongensis* GO16 as a platform for the *in vivo* synthesis of short and medium chain length polyhydroxyalkanoate blends. Bioresour Technol. 2023;387:129668. 10.1016/j.biortech.2023.129668 37572888

[bib12] Chiba S, Saito H et al. Human footprint in the abyss: 30 year records of deep-sea plastic debris. Mar Policy. 2018;96:204–12. 10.1016/j.marpol.2018.03.022

[bib13] Croucher NJ. Immune interface interference vaccines: an evolution-informed approach to anti-bacterial vaccine design. Microb Biotechnol. 2024;17:e14446. 10.1111/1751-7915.14446 38536702 PMC10970203

[bib14] Del Cerro C, Erickson E, Dong T et al. Intracellular pathways for lignin catabolism in white-rot fungi. Proc Natl Acad Sci USA. 2021;118:e2017381118. 10.1073/pnas.2017381118 33622792 PMC7936344

[bib15] Dodge AG, Thoma CJ, O'Connor MR et al. Recombinant *Pseudomonas* growing on non-natural fluorinated substrates shows stress but overall tolerance to cytoplasmically released fluoride anion. mBio. 2024;15:e02785–23. 10.1128/mbio.02785-23 38063407 PMC10790756

[bib16] Dupraz C, Reid RP, Braissant O et al. Processes of carbonate precipitation in modern microbial mats. Earth Sci Rev. 2009;96:141–62. 10.1016/j.earscirev.2008.10.005

[bib17] Falkowski PG, Fenchel T, Delong EF. The microbial engines that drive Earth’s biogeochemical cycles. Science. 2008;320:1034–9. 10.1126/science.1153213 18497287

[bib18] García-Franco A, Godoy P et al. Engineering styrene biosynthesis: designing a functional *trans*-cinnamic acid decarboxylase in *Pse u domonas*. Microb Cell Fact. 2024;23:69. 10.1186/s12934-024-02341-0 38419048 PMC10903017

[bib19] Gewin V. Microbiology must be represented at climate change talks. Nat Microbiol. 2023;8:2238–41. 10.1038/s41564-023-01534-4 38030905

[bib20] Gilbert JA, Blaser MJ, Caporaso JG et al. Current understanding of the human microbiome. Nat Med. 2018;24:392–400. 10.1038/nm.4517 29634682 PMC7043356

[bib21] Haas R, Nikel PI. Challenges and opportunities in bringing nonbiological atoms to life with synthetic metabolism. Trends Biotechnol. 2023;41:27–45. 10.1016/j.tibtech.2022.06.004 35786519

[bib22] Hernández-Arriaga AM, Campano C, Rivero-Buceta V et al. When microbial biotechnology meets material engineering. Microb Biotechnol. 2022;15:149–63. 10.1111/1751-7915.13975. 34818460 PMC8719833

[bib23] Hueso-Gil A, Calles B, de Lorenzo V. *In vivo* sampling of intracellular heterogeneity of *Pseudomonas putida* enables multiobjective optimization of genetic devices. ACS Synth Biol. 2023;12:1667–76. 10.1021/acssynbio.3c00009 37196337 PMC10278179

[bib24] IPCC. AR5 synthesis report: climate change 2014. 2014. https://www.ipcc.ch/report/ar5/syr/. (23 December 2023, date last accessed).

[bib25] IPCC. Global warming of 1.5°C. 2019. https://www.ipcc.ch/site/assets/uploads/sites/2/2019/06/SR15_Full_Report_High_Res.pdf.

[bib26] Jansson JK. Microorganisms, climate change, and the sustainable development goals: progress and challenges. Nat Rev Micro. 2023;21:622–3. 10.1038/s41579-023-00953-8 37500766

[bib27] Kalia VC, Patel SKS, Kwan Cho B et al. Emerging applications of bacteria as antitumor agents. Semin Cancer Biol. 2022;86:1014–25. 10.1016/j.semcancer.2021.05.012 33989734

[bib28] Kazemian N, Ramezankhani M, Sehgal A et al. The trans-kingdom battle between donor and recipient gut microbiome influences fecal microbiota transplantation outcome. Sci Rep. 2020;11:18349. 10.1038/s41598-021-82644-z PMC7591866 33110112

[bib29] Keasling J, Garcia Martin H, Lee TS et al. Microbial production of advanced biofuels. Nat Rev Microbiol. 2021;19:701–15. 10.1038/s41579-021-00577-w 34172951

[bib30] Li X, Gadar-Lopez AE, Chen L et al. Mining natural products for advanced biofuels and sustainable bioproducts. Curr Opin Biotechnol. 2023;84:103003. 10.1016/j.copbio.2023.103003 37769513

[bib31] López-García P, Moreira D. The symbiotic origin of the eukaryotic cell. C R Biol. 2023;346:55–73. 10.5802/crbiol.118 37254790

[bib32] Matilla MA, Monson RE, Murphy A et al. Solanimycin: biosynthesis and distribution of a new antifungal antibiotic regulated by two quorum-sensing systems. mBio. 2022;13:e02472–22. https.//doi.org/10.1128/mbio.02472-22 36214559 10.1128/mbio.02472-22 PMC9765074

[bib33] Merz JJ, Barnard P, Rees WE et al. World scientists’ warning: the behavioural crisis driving ecological overshoot. Sci Prog. 2023;106:00368504231201372. 10.1177/00368504231201372 37728669 PMC10515534

[bib34] Michán C, Blasco J, Alhama J. High-throughput molecular analyses of microbiomes as a tool to monitor the wellbeing of aquatic environments. Microb Biotechnol. 2021;14:870–85. 33559398 10.1111/1751-7915.13763 PMC8085945

[bib35] Moore CE, Meacham-Hensold K, Lemonnier P et al. The effect of increasing temperature on crop photosynthesis: from enzymes to ecosystems. J Exp Bot. 2021;72:2822–44. 10.1093/jxb/erab090 33619527 PMC8023210

[bib36] Nejman D, Livyatan I, Fuks G et al. The human tumor microbiome is composed of tumor type-specific intracellular bacteria. Science. 2020;368:973–80. 10.1126/science.aay9189 32467386 PMC7757858

[bib37] Pardo I, Calero P, Volke DC et al. A nonconventional archaeal fluorinase identified by *in silico* mining for enhanced fluorine biocatalysis. ACS Catal. 2022;12:6570–7. doi.org/10.1021/acscatal.2c01184 35692250 10.1021/acscatal.2c01184 PMC9173684

[bib38] Pozo de la Hoz J, Rivero J, Azcón-Aguilar C et al. Mycorrhiza-induced resistance against foliar pathogens is uncoupled of nutritional effects under different light intensities. J Fungi. 2021;7:402. 10.3390/jof7060402 PMC8224078 34063889

[bib39] Ramos JL, Duque E. Twenty-first-century chemical odyssey: fuels versus commodities and cell factories versus chemical plants. Microb Biotechnol. 2019;12:200–9. 10.1111/1751-7915.13379 30793487 PMC6389845

[bib40] Rappuoli R, Young P, Ron E et al. Save the microbes to save the planet. A call to action of the International Union of the Microbiological Societies (IUMS). One Health Outlook. 2023;5:5. 10.1186/s42522-023-00077-2 36872345 PMC9986037

[bib41] Rassmusen S, Bedau MA., Chan L, Deamer D, Krakauer DC, Packard NH, Stadler PF (eds). Protocells: Bridging Nonliving and Living Matter. Cambridge, Massachusetts: The MIT Press, 2009.

[bib42] Ripple WJ, Wolf C, Gregg JW et al. World scientists’ warning of a climate emergency. Bioscience. 2022;72:1149–55. 10.1093/biosci/biac083

[bib43] Ripple WJ, Wolf C, Gregg JW et al. The 2023 state of the climate report: entering uncharted territory. Bioscience. 2023;73:841–50. 10.1093/biosci/biad080

[bib44] Roca A, Matilla MA. Microbial antibiotics take the lead in the fight against plant pathogens. Microb Biotechnol. 2023;16:28–33. 36464960 10.1111/1751-7915.14185 PMC9803328

[bib45] Salvachúa D, Rydzak T, Auwae R et al. Metabolic engineering of *Pseudomonas putida* for increased polyhydroxyalkanoate production from lignin. Microb Biotechnol. 2020;13:290–8. 10.1111/1751-7915.13481 31468725 PMC6922519

[bib46] Sánchez-Baracaldo P, Bianchini G, Wilson JD et al. Cyanobacteria and biogeochemical cycles through Earth history. Trends Microbiol. 2022;30:143–57. 10.1016/j.tim.2021.05.008 34229911

[bib47] Shears J. Is there a role for synthetic biology in addressing the transition to a new low-carbon energy system?. Microb Biotechnol. 2019;12:824–7. 10.1111/1751-7915.13462 31342652 PMC6680603

[bib48] Sheehan PM, Harris MT. Microbialite resurgence after the Late Ordovician extinction. Nature. 2004;430:75–78. 15229600 10.1038/nature02654

[bib49] Singh BK, Delgado-Baquerizo M, Egidi E et al. 2023a. Climate change impacts on plant pathogens, food security and paths forward. Nat Rev Microbiol. 21:640–56. 10.1038/s41579-023-00900-7 37131070 PMC10153038

[bib50] Singh BK, Fraser ED, Arnold T et al. Food systems transformation requires science–policy–society interfaces that integrate existing global networks and new knowledge hubs. Nat Food. 2023b;4:1–3. 10.1038/s43016-022-00664-y. 37118566

[bib51] Singh BK, Yan ZZ, Whittaker M et al. Soil microbiomes must be explicitly included in One Health policy. Nat Microbiol. 2023c;8:1367–72. 10.1038/s41564-023-01386-y 37268853

[bib52] Somerville C, Youngs H, Taylor C et al. Feedstocks for lignocellulosic biofuels. Science. 2010;329:790–2. 10.1126/science.1189268 20705851

[bib53] Sun X, Wang J, Dong M et al. Food spoilage, bioactive food fresh-keeping films and functional edible coatings: research status, existing problems and development trend. Trends Food Sci Technol. 2022;119:122–32.

[bib54] Timmis K. Microbiology education: a significant path to sustainably improve the human and biosphere condition. Microlife. 2023a;4:uqad013. 10.1093/femsml/uqad013 37223743 PMC10117706

[bib55] Timmis K. A road to microbiology literacy (and more): an opportunity for a paradigm change in teaching. J Microbiol Biol Educ. 2023b;24:e00019–23. 10.1128/jmbe.00019-23 37089226 PMC10117087

[bib56] Timmis K, Ramos JL. The soil crisis: the need to treat as a global health problem and the pivotal role of microbes in prophylaxis and therapy. Microb Biotechnol. 2021;14:769–97. 10.1111/1751-7915.13771 33751840 PMC8085983

[bib57] United Nations. 2023. The sustainable development goals report. Special Edition. www.unstasts.un.org

[bib58] Valdivia M, Galán JL, Laffarga J et al. Biofuels 2020: biorefineries based on lignocellulosic materials. Microb Biotechnol. 2016;9:585–94. 10.1111/1751-7915.12387 27470921 PMC4993176

[bib59] Wu M, Wang H. Re-considering and designing microbiomes for future waste biorefinery. Microb Biotechnol. 2024;17:e14395. 10.1111/1751-7915.14395 38206186 PMC10835331

